# Image-guided *in-Vivo* Needle-Based Confocal Laser Endomicroscopy in the Prostate: Safety and Feasibility Study in 2 Patients

**DOI:** 10.1177/15330338221093149

**Published:** 2022-07-05

**Authors:** Luigi A.M.J.G. van Riel, Abel Swaan, Christophe K. Mannaerts, Rob A.A. van Kollenburg, C. Dilara Savci Heijink, Theo M. de Reijke, Daniel M. de Bruin, Jan Erik Freund

**Affiliations:** 1 Department of Urology, 26066 Amsterdam University Medical Centers, University of Amsterdam, Amsterdam, the Netherlands; 2 Department of Biomedical Engineering and Physics, Amsterdam University Medical Centers, University of Amsterdam, Amsterdam, the Netherlands; 3 Department of Pathology, 26066 Amsterdam University Medical Centers, University of Amsterdam, Amsterdam, the Netherlands

**Keywords:** confocal laser endomicroscopy (non-MESH), prostate cancer (MESH), diagnosis (MESH), Gleason (MESH), biopsy (MESH)

## Abstract

**Purpose:** To assess the safety and technical feasibility of *in-vivo* needle-based forward-looking confocal laser endomicroscopy in prostate tissue. **Methods:** For this feasibility study, 2 patients with a suspicion of prostate cancer underwent transperineal needle-based confocal laser endomicroscopy during ultrasound-guided transperineal template mapping biopsies. After intravenous administration of fluorescein, needle-based confocal laser endomicroscopy imaging was performed with a forward-looking probe (outer diameter 0.9 mm) in 2 trajectories during a manual push-forward and pullback motion. A biopsy was taken in a coregistered parallel adjacent trajectory to the confocal laser endomicroscopy trajectory for histopathologic comparison. Peri- and postprocedural adverse events, confocal laser endomicroscopy device malfunction and procedural failures were recorded. Needle-based confocal laser endomicroscopy image quality assessment, image interpretation, and histology were performed by an experienced confocal laser endomicroscopy rater and uro-pathologist, blinded to any additional information. **Results:** In both patients, no peri- and post-procedural adverse events were reported following needle-based confocal laser endomicroscopy. No confocal laser endomicroscopy device malfunction nor procedural failures were reported. Within 1.5 min after intravenous administration of fluorescein, needle-based confocal laser endomicroscopy image quality was sufficient for interpretation for at least 14 min, yielding more than 5000 confocal laser endomicroscopy frames per patient. The pullback confocal laser endomicroscopy recordings and most of the push-forward recordings almost only visualized erythrocytes, being classified as non-representative. During the push-forward recordings, prostate tissue was occasionally visualized in single frames, insufficient for histopathologic comparison. Prostate carcinoma was identified by biopsy in one patient (Gleason score 4 + 3 = 7, >50%), while the biopsy from the other patient showed no malignancy. **Conclusion:** Needle-based confocal laser endomicroscopy imaging of *in-vivo* prostate tissue with a forward-looking confocal laser endomicroscopy probe is safe without device malfunctions or procedural failures. Needle-based confocal laser endomicroscopy is technically feasible, but the acquired confocal laser endomicroscopy datasets are non-representative. The confocal laser endomicroscopy images’ non-representative nature is possibly caused by bleeding artifacts, movement artifacts and a lack of contact time with the tissue of interest. A different confocal laser endomicroscopy probe or procedure might yield representative images of prostatic tissue.

## Introduction

Prostate cancer (PCa) is the most commonly diagnosed malignancy in men and the second cause of cancer-related deaths for men worldwide.^1,2^ Focal therapy is explored as an alternative treatment for low- and intermediate-risk PCa. This treatment focuses on targeting the malignant prostate tissue while sparing nearby tissues to improve functional outcomes.^3,4^ Identification and demarcation of clinically significant PCa are, therefore, essential to focal therapy.

Currently, transrectal ultrasound (TRUS) and prostate MRI are used to detect suspected lesions.^5^ Yet, the sensitivity of TRUS is low.^6,7^ Also, the systematic 12-core TRUS guided biopsy scheme has a high false-negative rate of 20% to 30% with a PCa detection rate of approximately 65%.^8,9^ The addition of prostate MRI-based targeted biopsies improved the clinically significant PCa detection rate in men with a suspicion of PCa.^10,12^ However, prostate MRI targeted biopsy is limited by the voxel size of MRI, a moderate interreader reproducibility and an unstandardized targeting method. Furthermore, TRUS and prostate MRI lack information on a microscopic level. Therefore, histopathology remains the gold standard for diagnostics. However, the standard histopathology work-up is time-consuming, labor-intensive and offline.^13^ Real-time imaging at cellular level could overcome the aforementioned limiting factors, potentially reducing the number of biopsies.

As a matter of fact, probe-based confocal laser endomicroscopy (CLE) is a novel fluorescent contrast-based optical imaging technique that enables real-time visualization of tissue structures at a cellular level. The use of CLE for *in-vivo* identification and grading of cancer has been proven successful in hollow organs.^14,16^ Recently, Lopez *et al* demonstrated the feasibility of *in-vivo* CLE imaging the neurovascular bundle during robot-assisted radical prostatectomy (RARP).^17^ Furthermore, *ex-vivo* CLE images of surgical prostate specimens were coregistered with histopathology to identify benign prostatic glands, PCa glands and extracapsular extension of cancer. CLE has also been applied in a needle-based fashion to identify lung tumors and lymphatic metastases.^18,19^ Concerning the promising results of *in-vivo* needle-based CLE (nCLE) in lung cancer, we hypothesized that nCLE might also be suited for identifying and demarcating PCa adjunct to prostate biopsies.

Therefore, as previously published in a protocol paper, this study assesses the technical feasibility and safety of *in-vivo* nCLE during transperineal prostate biopsies.^20^ Additionally, acquired nCLE data are compared with histology from colocalized biopsies. This study follows the IDEAL phase 2a for the assessment of novel techniques in a surgical environment.^21^

## Materials and Methods

As published previously, this study is an investigator-initiated, prospective *in-vivo* feasibility study, evaluated by the local institutional review board of the Amsterdam University Medical Centers under registry number “NL57326.018.17” and consecutive approval number “2017_130#B2017389.”^20^ The study is registered on clinicaltrails.gov as Focal Prostate Imaging with CLE and OCT (FPI) (NCT03253458).

Patients with a clinical suspicion of PCa and a planned transperineal template mapping biopsy (TTMB) of the prostate were eligible, aiming to include 2 patients. TTMB was planned for diagnostic work-up for focal therapy selection. Participants gave written informed consent.

**Figure 1.**
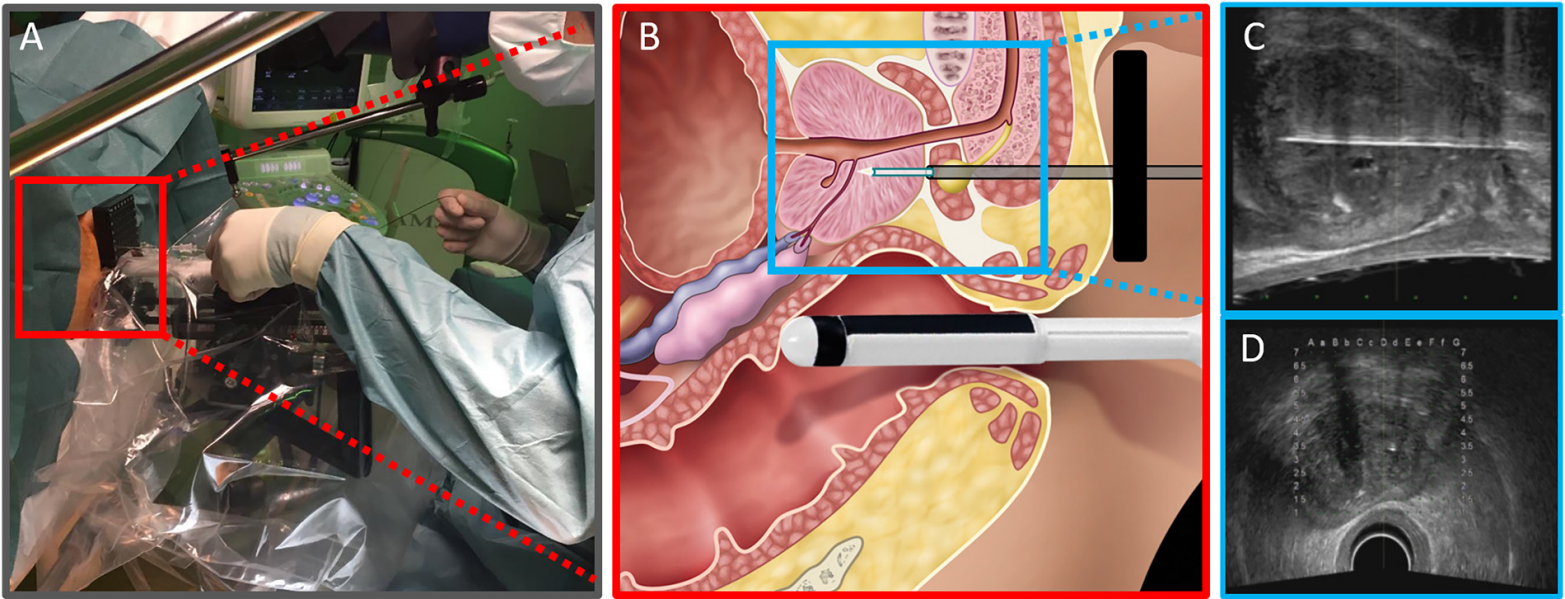
(A) An overview of a TTMB setting in the operating room including (B) a CLE measurement of the prostate with a forward imaging Cellvizio® AG-Flex 19 fiber optic mini probe-based system under transrectal ultrasound guidance using (C) sagittal and (D) axial planes. It was adapted from “The First In Vivo Needle-Based Optical Coherence Tomography in Human Prostate: A Safety and Feasibility Study” by Swaan *et al*, 2019, Lasers in surgery and medicine.

nCLE was performed during a TTMB procedure (Figure 1) using the AG-Flex 19 fiber optic probe-based system with 0.9 mm probe diameter, 325 µm field of view, and a resolution of 3.5 µm (Cellvizio® System, Mauna Kea Technologies). The fluorescent light from the contrast-stained tissue at 40 to 70 μm from the lens is focused through a pinhole to generate high-resolution images of the tissue.

Standard TTMB was performed in the operating room with patients in a lithotomy position. A TRUS scanner (VISION Preirus®; Hitachi Medical Systems) with the biplane probe (EUP-U533; Hitachi Medical Systems) and endocavity balloon were used for TTMB and CLE probe guidance. Preoperative prostate MRI consisted of at least a minimum of T1-weighted, T2-weighted, diffusion-weighted imaging (DWI) and calculation of apparent diffusion coefficient (ADC) maps. MRI series were evaluated by dedicated uroradiologists according to prostate imaging-reporting and data system (PI-RADS) version 2.^22^ For each patient, 2 nCLE trajectories were assigned prior to imaging. Based on the PCa suspicious lesions on MRI (PI-RADS 4 or 5), a “suspicious trajectory” for nCLE imaging was assigned. The suspicious prostate tissue was targeted using a cognitive fusion of the MRI and real-time TRUS images. The second trajectory was targeted at benign prostate tissue (“benign trajectory”). Under ultrasound guidance, a 17-gauge trocar needle was introduced, reaching into the region of interest. Subsequently, the inner part of the trocar needle was removed and the CLE probe was introduced. The outer part of the trocar needle was retracted while the CLE probe remained in the region of interest and generated static CLE images before fluorescein injection (Figure 1).

**Figure 2.**
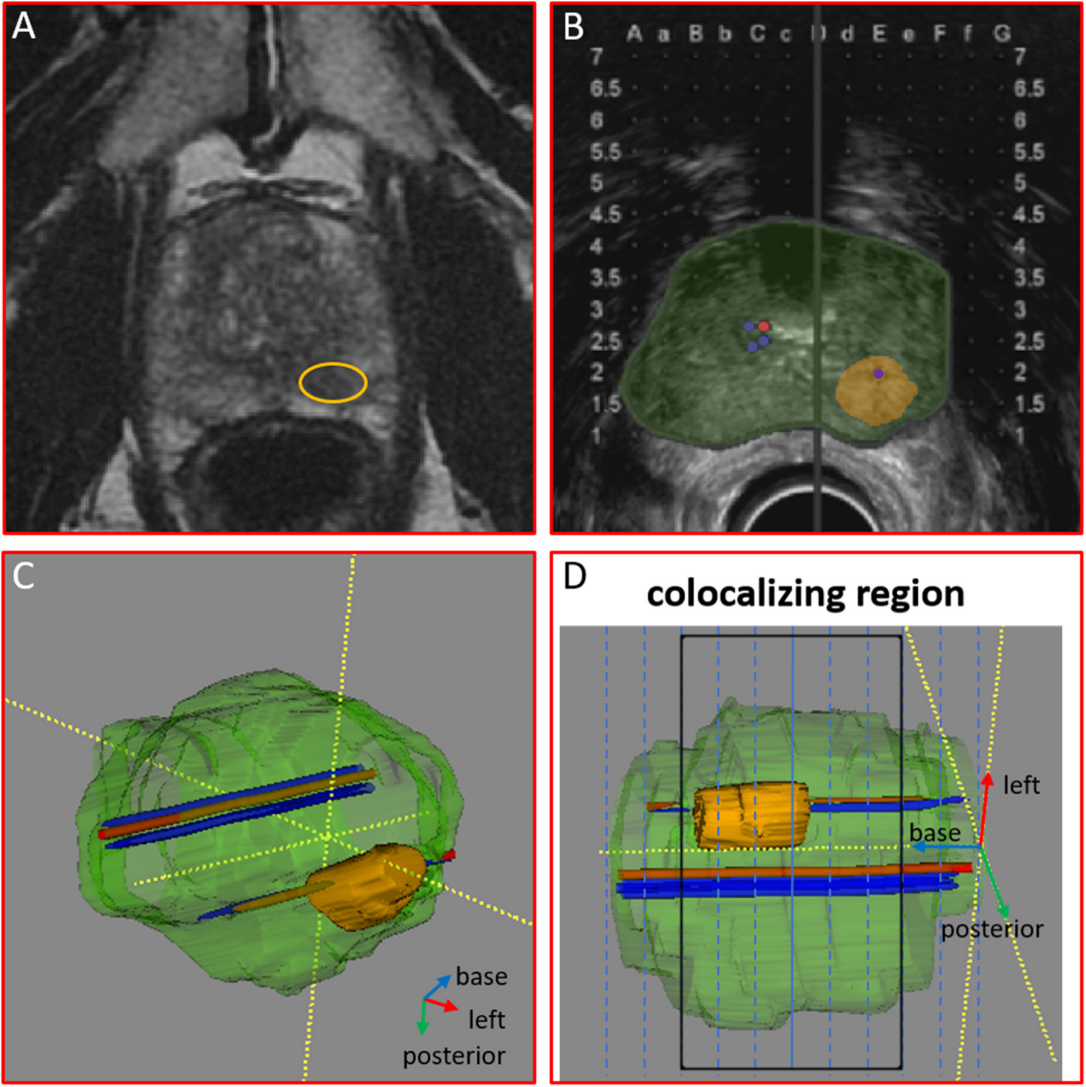
A 3-dimensional (3D) model of the prostate based on fused segmentations of prostate MRI and TRUS images. (A) Axial T2-weighted prostate MRI and (B) TRUS images were used for segmentation of the prostate and tumor (orange). (C) Registered trajectories of CLE measurements (red) and biopsies (blue) were annotated). Elastic fusion of prostate MRI and TRUS segmentations of the prostate, tumor, and trajectories resulted in a 3D model. (D) Corresponding regions of CLE measurement and biopsy trajectories were identified and used for coregistration of CLE images with histopathology.

A single bolus of 2.5 mL of 10% sodium fluorescein (Fresenius Kabi) was administered intravenously for staining of the extracellular matrix before imaging nCLE (521 nm emission wavelength).^23^ Subsequently, nCLE imaging was performed under TRUS guidance during a manual push-forward and pullback motion in both assigned trajectories. CLE images were recorded at a scan rate of 12 frames per second. Next, a standard biopsy core was taken parallel to the imaged trajectories by combining the same grid coordinates and the ultrasound image (Figure 2B).

The nCLE trajectories and the biopsy locations (Figure 2) were registered with the 3D ultrasound registration software (3DBiopsy®, Inc) for offline comparison. Lastly, the regular TTMB procedure was performed, using an 18G biopsy needle.

Adverse events during the procedure and follow-up were registered according to the Clavien-Dindo classification.^24^ Technical feasibility was determined upon CLE device malfunction, procedural failures, and CLE data quality.

Segmentation of preoperative axial T2-weighted prostate MRI and perioperative TRUS images with the nCLE and biopsy trajectories was performed using ITK-SNAP software.^25^ This enabled the construction of an interactive 3D model of the prostate with its suspicious lesions and the nCLE and biopsy trajectories (Figure 2C and D).

Assessment of nCLE image quality and image interpretation was performed offline by an experienced CLE rater, blinded to any clinical and histopathologic information (J.F.). CLE images were analyzed according to the previously described CLE characteristics of prostate tissue and the previous experience with CLE assessment of urothelial carcinoma and lung cancer.^14,17,18^ The histopathologic assessment was performed according to the standard clinical care by an experienced uropathologist, blinded to CLE data (D.S.).

## Results

### Patient Characteristics

Patient 1 was 71 years old with a prostate volume of 60 mL, a PSA value of 12.1 ng/mL, suspicious DRE, and PI-RADS 5 lesion at the posterolateral peripheral zone in the right apex.

Patient 2 was 55 years old with a prostate volume of 44 mL, a PSA value of 21.4 ng/mL, benign DRE, and a PI-RADS 4 lesions of 12 mm at the posterolateral peripheral zone in the left mid prostate (Figure 2A and B).

### Safety and Feasibility

CLE imaging was performed without any peri- or post-procedural adverse events. The intravenous fluorescein injection led to a flash of contrast within 90 to 100 s after injection (wash-in phase). This was classified as an adequate visible contrast and sufficient for CLE image interpretation, based on the nCLE operators’ experience. nCLE imaging with adequate contrast was feasible for at least 14 min. In order to minimalize patient burden, CLE imaging was not continued until the wash-out phase of the contrast agent was finished. Following a single fluorescein injection in both patients, enabling nCLE imaging of both “benign” and “suspicious trajectories.” Two push-and-image and 2 pullback-and-image datasets were acquired in patient 1, yielding a total of 6030 out of 7230 frames with adequate contrast. Four pullback-and-image and 2 push-and-image datasets were acquired in patient 2, yielding a total of 5186 out of 6521 frames with adequate contrast. Overall extra time for the nCLE procedure was 14 min for patient 1 and 17 min for patient 2. No device malfunction or procedural failures occurred during nCLE imaging.

nCLE was technically feasible, yielding CLE images with an adequate fluorescent signal. It was noted that the manual pullback-and-scan method could be conducted at a continuous movement, opposed to the irregular movement of the push-and-image method.

### Coregistration of CLE Datasets and Biopsy Cores

Biopsy locations were coregistered with nCLE trajectories and showed that nCLE trajectories were approximated by core needle biopsies in a parallel fashion (Figure 2C and D).

### Histological Analysis

All core needle biopsies (n = 32) of patient 1 showed prostatic tissue without malignancy. Patient 2 had unilateral left adenocarcinoma of the prostate (Gleason score 4 + 3 = 7 with cribriform growth) in 7 out of 30 core needle biopsies originating from the peripheral zone in the apex, mid, and base of the prostate. The biopsy parallel to the “suspicious trajectory” showed adenocarcinoma of the prostate (Gleason Score 4 + 3 = 7, cribriform growth present) with a tumor volume of 50%. There was no malignancy in the biopsy parallel to the “benign trajectory” in patient 2.

### CLE Dataset Analysis

**Figure 3.**
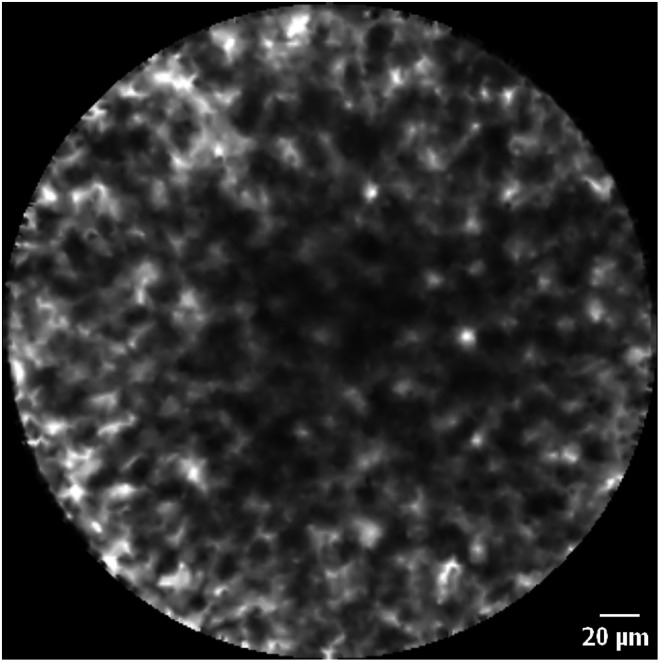
nCLE image of discohesive cells, being identified as erythrocytes due to bleeding.

**Figure 4.**
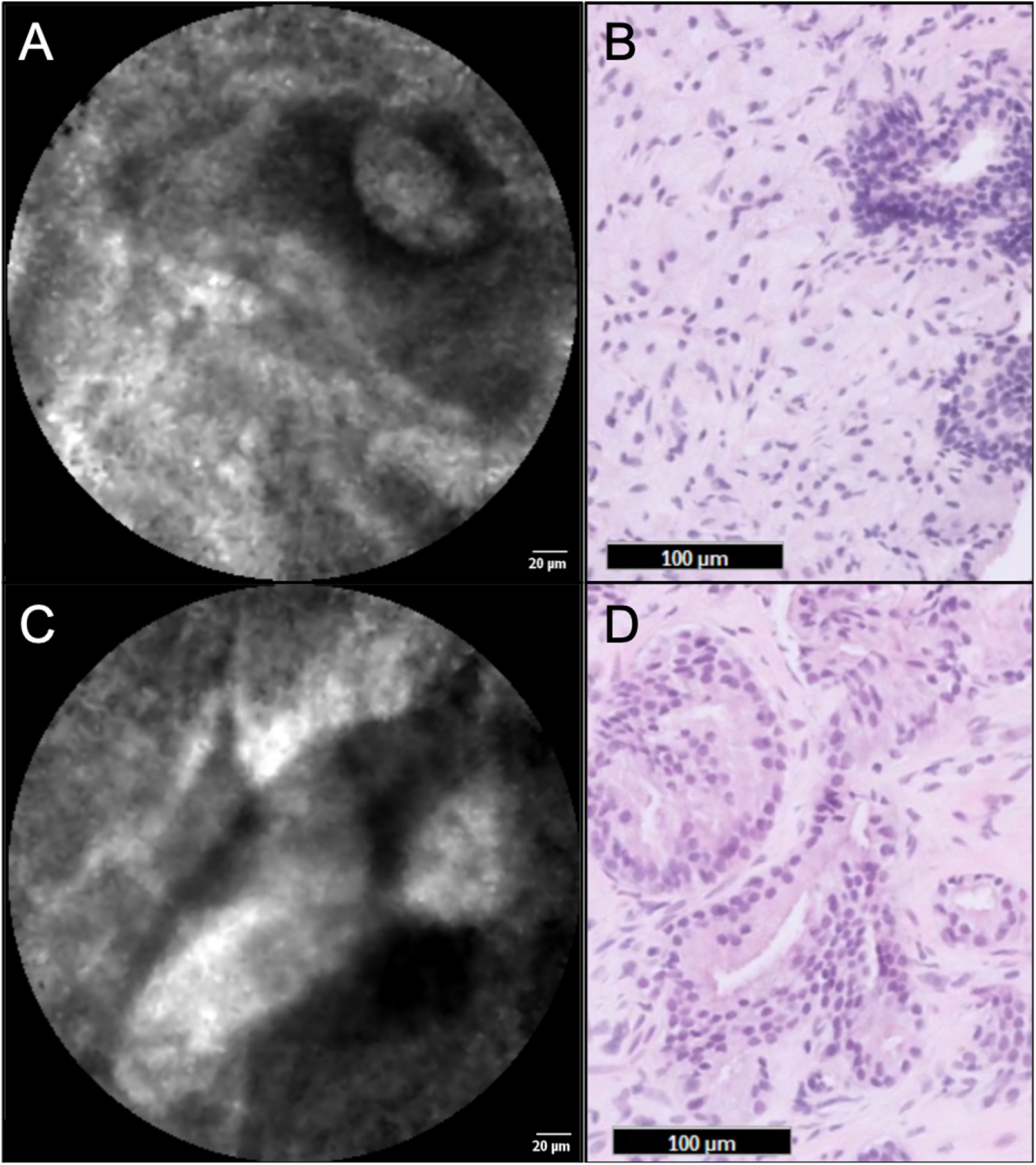
nCLE image of prostatic tissue (A + C) and similarly appearing benign prostate glands and stroma in H&E stained histology slides (B + D), obtained from parallel trajectories, from the patient with Gleason score 4 + 3 = 7 prostate cancer in core needle biopsies.

The pullback-and-image CLE datasets consisted of only discohesive cells’ images, being identified as erythrocytes from bleeding (Figure 3). These CLE datasets were classified as non-representative. From the push-and-image recordings, only in individual frames, structures that may resemble prostate glands and stroma were identified (Figure 4). Differentiation between benign or malignant glands was not readily feasible. Colocalization of the identified structures with histopathology was not feasible due to insufficient CLE image frames with identifiable prostate tissue. Moreover, the CLE image frames with structures that may resemble prostate tissue were of low quality due to movement artifacts and admixture of erythrocytes from bleeding.

## Discussion

This study showed that nCLE of the prostate with a forward-looking CLE probe is safe and technically feasible. However, nCLE did not yield diagnostic value images with the present protocol, nor did the approach allow for histopathologic comparison. The acquired CLE datasets almost exclusively visualized blood. Structures that may resemble prostatic structures were only identifiable in a low number of individual frames from the push-and-image approach. Despite sufficient fluorescent signal, the frames’ image quality with recognizable structures was poor, most likely due to movement artifacts and admixture of erythrocytes from bleeding. In short, nCLE, acquired with a forward-looking CLE probe with the present protocol, is not applicable for adequate prostate tissue identification.

The non-representative nature of the investigated nCLE can be explained by the forward-looking CLE probe's continuous movement and, hence, the limited contact with the tissue of interest. Moreover, the lack of contact with the tissue of interest enables the accumulation of erythrocytes in the CLE probe's focal plane. Wijmans *et al* also reported that a pullback-and-image led to obscured imaging caused by bleeding during forward-looking nCLE in lymph nodes.^18^ These factors hamper the image quality and the acquisition of sufficient image frames of the region of interest.

In comparison with the reported *ex-vivo* CLE images by Lopez *et al* and our previous *ex-vivo* CLE images, the image quality of the *in-vivo* nCLE images of the structures that resemble prostate tissue is of inferior quality, especially owing to the nature of the CLE probe and bleeding artifacts.^17,20^ The CLE probe for the needle-based approach (outer probe diameter 0.9 mm) lacks the ultra-high-definition (UHD) lens system of the UHD-R CLE probe, resulting in a 3.5 ×  higher lateral resolution. The UHD-R CLE probe, used for *ex-vivo* imaging by Lopez *et al*, however, is not suitable for a needle-based approach due to its outer probe diameter of 2.6 mm. Moreover, in the current set-up with a forward-looking CLE probe, an even higher resolution will possibly not be beneficial due to the aforementioned limited tissue contact and bleeding and movement artifacts.

A procedural limitation of the current approach is the acquisition of CLE datasets perpendicular to the core needle biopsies. This limitation is caused by the forward-looking nature of the CLE probe, complicating one-to-one histopathologic comparison, which is needed to create an atlas of different prostate tissue characteristics. The technical and procedural drawbacks might be overcome with a modified CLE probe. One option could be a sideward-looking CLE probe with an automated rotary pullback system and a flushing channel, similar to the optical coherence tomography (OCT) probe used for *in-vivo* prostate imaging.^26^ This would result into increased dataset volume, consistent pullback velocity and most optimal comparison with biopsies. To our knowledge, however, a sideward-looking CLE probe is not under development yet. Therefore, another option might be a forward-looking CLE probe with a flushing channel and with the focal plane further away from the tip of the probe, so that accumulated blood in front of the probe will be out of focus.

Interestingly, in this study, nCLE imaging of the suspicious trajectory at the peripheral zone was to be compared with the benign trajectory at the transitional zone in the prostate. One could argue that tissue characteristics of these zones are intrinsically different, which could complicate diagnostic evaluation of nCLE imaging. However, nCLE images were compared with histology, as a reference standard. Based on histologic criteria, there are no differences in adenocarcinoma located in the transitional zone and peripheral zone. This also applies for benign epithelial structures found in the transitional zone and the peripheral zone. Therefore, for the purpose of this study, it was found to be a robust comparison.

A significant limitation of CLE for PCa diagnosis might be the lack of visualization of cytonuclear features. The applied fluorescein for CLE imaging does not penetrate the cells. Therefore, CLE only visualizes the extracellular matrix. As a result, the diagnostic accuracy of CLE for adenocarcinoma of the prostate without major architectural changes (Gleason pattern 3) is limited.

Nevertheless, as Lopez *et al* reported, *ex-vivo* CLE imaging with a forward-looking probe might enable the detection of prostate tissue and adenocarcinoma.^17^ Moreover, the diagnostic ability of nCLE has been proven in other organs.^18,27^ For example, nCLE data have been reported in mediastinal lymph nodes for lung cancer detection using a push-and-pull technique with an identical forward-looking probe, yielding a sensitivity and specificity of 90% and 89%, respectively.^18^

Accurate prostate imaging is crucial to minimize biopsy procedures, increase significant PCa detection, reduce insignificant PCa detection, and provide a reliable follow-up method for active surveillance and focal therapy.^28,31^ Optical imaging techniques, like CLE, may provide visualization of prostate tissue for real-time cancer detection. Incorporating CLE into the diagnostic workup of PCa could serve as an adjunct to MRI/US-fusion biopsy, reduce the number of biopsy cores, and histopathologic workload. Moreover, CLE could support the see-and-treat principle of focal therapy using the same optical fiber.^32^ With these potential benefits in mind, further efforts should be made to advance nCLE of the prostate, starting with the modification of the procedural approach with the forward-looking probe or with the development of a sideward-looking probe. Merging OCT and CLE into a single sideward-looking probe with dual imaging modalities may additionally augment both techniques’ diagnostic potential.

## Conclusion

*In-vivo* needle-based forward-looking CLE is safe without device malfunctions or procedural failures. nCLE is technically feasible, but the acquired CLE datasets are non-representative. The CLE images’ non-representative nature is possibly caused by bleeding artifacts, movement artifacts and a lack of contact time with the tissue of interest. In short, CLE images, acquired in a needle-based fashion with a forward-looking CLE probe during a continuous push or pull movement are not applicable for prostate tissue detection.

## Authors’ Note

Luigi A.M.J.G. van Riel and Abel Swaan contributed equally. This study is approved by the Amsterdam Unversity Medical Centers institutional review board under registry number: NL57326.018.17. The date of approval was July 7, 2017. The approval identification code is: 2017_130#B2017389. Clinical trials: Focal Prostate Imaging With CLE and OCT (FPI); NCT03253458. https://clinicaltrials.gov/ct2/show/NCT03253458. This trial is registered in the Dutch Toetsingonline trial registration system, number: NL 57326.018.17 (https://www.toetsingonline.nl/).

## Abbreviations

CLE: confocal laser endomicroscopy
DRE: digital rectal examination
MRI: magnetic resonance imaging
nCLE: *in-vivo* needle-based forward-looking CLE
OCT: optical coherence tomography
PCa: prostate cancer
PI-RADS: Prostate Imaging Reporting Data System
TRUS: transrectal ultrasound
TTMB: transperineal template mapping biopsy
